# Doubling down on forensic twin studies

**DOI:** 10.1371/journal.pgen.1007831

**Published:** 2018-12-20

**Authors:** Gregory P. Copenhaver, Bruce Weir, Mark Rothstein, Hua Tang, Scott M. Williams, Gregory S. Barsh

**Affiliations:** 1 Department of Biology and the Integrative Program for Biological and Genome Sciences, University of North Carolina at Chapel Hill, Chapel Hill, North Carolina, United States of America; 2 Department of Biostatistics, University of Washington, Seattle, Washington, United States of America; 3 Institute for Bioethics, Health Policy and Law, University of Louisville School of Medicine, Louisville, Kentucky, United States of America; 4 Department of Genetics, Stanford University School of Medicine, Stanford, California, United States of America; 5 Departments of Population and Quantitative Health Sciences, Institute of Computational Biology, Case Western Reserve University, Cleveland, Ohio, United States of America; 6 HudsonAlpha Institute for Biotechnology, Huntsville, Alabama, United States of America

Twins have amazed and beguiled the human imagination throughout human history. In Genesis, Jacob uses his status as a twin to deceive his aged father Isaac and steal his bother Esau’s birthright—an event that sets the stage for the rest of the Old Testament. In Shakespeare’s *The Comedy of Errors*, not one, but two, sets of twins (Dromio and Antipholus × 2) are the core of the bard’s tale.

The resemblance between monozygotic (identical) twins is not only a source of wonder but also grist for genetic and genomic studies because distinguishing one twin from another can be challenging to even close family members. This task—distinguishing the identities of a monozygotic twin pair—is also a challenge in many practical situations, from airport security to criminal investigation. In the accompanying article, Krawczak and colleagues describe an analytical approach for using DNA sequencing data to identify which individual, from a set of monozygotic twins, matches a previously obtained sample [1]. This approach is a compelling application of genetic principles and will be an important tool for a range of forensic questions. Nonetheless, evaluation of their manuscript prompted a vigorous discussion at the editorial level, and that dialogue played a significant role in shaping the form of the published paper. We believe an open description of that process will help frame the manuscript as well as provide an instructive case study of issues that lie at the intersection of human genetics and society.

The manuscript submitted initially by Krawczak and colleagues had two distinct components. The first was the analytical framework that now comprises the majority of the published paper. The second was a proof-of-principle application of that framework to monozygotic twins in two forensic settings—one a criminal investigation and the other a civil legal proceeding. In both cases, the authors, acting in their capacity as a forensic analysis lab, followed established ethical protocols. Sample collection was authorized by the appropriate legal and institutional authorities, and subjects were informed of the analysis that would be performed. The initial version of the manuscript was associated with a statement from an Institutional Review Board (IRB) that recent European Union (EU) data protection legislation would (1) allow processing of personal data for research and scientific purposes other than those for which the data were initially collected, and (2) because the data contained in the manuscript would be insufficient to allow identification of the subjects, prior consent by the subjects was not required. To be clear, this IRB approval was independent of, and in addition to, the authorization to collect and analyze the original samples. Therefore, the authors were diligent in seeking and obtaining the necessary permissions at each stage of their work.

Nonetheless, the manuscript elicited discussion among the *PLOS Genetics* editors for two reasons. First, as with any study that deals with human material, we were concerned about the potential risks of personally identifying the subjects. Although we agreed with the IRB that the very small amount of DNA sequence data in the manuscript posed no risk by itself of subject identification, knowledge of the case details necessary to justify their inclusion in the study posed a substantial risk of identification (or misidentification) and had the potential to compromise the privacy of the individuals involved.

The *PLOS Genetics* editors were also concerned about the autonomy and potential vulnerability of individuals to whom the framework presented in the manuscript was being applied. Even though these individuals were informed that their DNA samples would be used for the purposes of forensic identification, communication as a responsibility of adherence to legal requirements is fundamentally different from informed consent to participate in research. In general, we believe that the potential impact of all genetic research is strengthened when an application of that research to a biological question or an important societal problem can be described along with the research. In this instance, however, the cases included members of unique classes of subjects, i.e., subject of criminal and/or civil litigation. Therefore, we believed that even in the absence of concerns regarding privacy, publishing a research article that described specific and real cases would risk compromising principles of social justice and risk failing to appropriately respect autonomy of subjects in making a meaningful choice.

Parallel to the aforementioned ethical concerns, the manuscript had been successfully peer-reviewed for scientific accuracy and significance. In balancing the societal benefits of a promising analytical framework for forensic DNA investigations versus our responsibilities as editors and scientists, we decided to offer publication of the manuscript but only after the proof-of-principle cases had been removed. Krawczak and colleagues agreed to these terms but in doing so noted that they imposed an important cost. As originally formulated, the manuscript clearly described how prior standard analyses were insufficient to enable their lab to differentiate between forensic samples from monozygotic twins and how this gap in analytical ability motivated the development of their new framework. As editors, we recognize and appreciate the importance and power of conveying the story behind the research. We also recognize that in asking the authors to restructure their manuscript we risk diminishing the potential impact of the work. As a result, we are grateful to Krawczak and colleagues for working with us to find a path forward that met their needs as authors, satisfied ours as a journal, and most importantly, respects the central tenets of biomedical ethics.
